# A Comparative Study of Tests for Homogeneity of Variances with Application to DNA Methylation Data

**DOI:** 10.1371/journal.pone.0145295

**Published:** 2015-12-18

**Authors:** Xuan Li, Weiliang Qiu, Jarrett Morrow, Dawn L. DeMeo, Scott T. Weiss, Yuejiao Fu, Xiaogang Wang

**Affiliations:** 1 Department of Mathematics and Statistics, York University, 4700 Keele Street, Toronto, ON, M3J1P3, Canada; 2 Channing Division of Network Medicine, Brigham and Women's Hospital, Harvard Medical School, 181 Longwood Avenue, Boston, MA, 02115, United States of America; CEA - Institut de Genomique, FRANCE

## Abstract

Variable DNA methylation has been associated with cancers and complex diseases. Researchers have identified many DNA methylation markers that have different mean methylation levels between diseased subjects and normal subjects. Recently, researchers found that DNA methylation markers with different variabilities between subject groups could also have biological meaning. In this article, we aimed to help researchers choose the right test of equal variance in DNA methylation data analysis. We performed systematic simulation studies and a real data analysis to compare the performances of 7 equal-variance tests, including 2 tests recently proposed in the DNA methylation analysis literature. Our results showed that the Brown-Forsythe test and trimmed-mean-based Levene's test had good performance in testing for equality of variance in our simulation studies and real data analyses. Our results also showed that outlier profiles could be biologically very important.

## Introduction

DNA methylation has been identified as a regulator of human gene expression. DNA methylation suppresses the expression of endogenous retroviral genes and other harmful stretches of DNA that have been incorporated into the host genome over time. Variable DNA methylation has been associated with cancers and many complex diseases. One possible epigenetic mechanism for the occurrence of a cancer or other complex diseases is that methylation of a subset of DNA methylation markers is modified by both environmental factors and genetic factors and in turn the variable DNA methylation modifies the expression of genes which may eventually leads to the occurrence of the disease. Hence, it is important to detect the subset of DNA methylation markers that are associated with the disease of interest to eventually achieve the goal of disease prevention and precision therapeutics.

Highthroughput DNA methylation data are now available to help researchers to measure methylation levels of tens of thousands of DNA methylation markers (e.g. CpG sites) at a time. Hence, researchers can efficiently evaluate which of these markers are differentially methylated between diseased subjects and non-diseased subjects.

Usually, researchers are interested in testing equality of mean DNA methylation level among different groups of subjects. Recently, several researchers [1,2,3,4,5,6] reported that CpG sites that are differentially variable between diseased subjects and non-diseased subjects also play important roles in uncovering the underlying mechanisms of complex diseases. These researchers applied the classic F test or equivalent Bartlett's test [7] to test if the variances between 2 groups of subjects are the same or not.

It is well-known that the F test/Bartlett's test is sensitive to the departure of the normality assumption and is sensitive to outliers. More than 50 tests have been proposed in the statistical literature to improve the F test/Bartlett's test. Conover et al. (1981)[8] compared 56 equal-variance testing procedures using simulation studies. The Brown-Forsythe test[9] was one of the top performers in Conover et al.'s (1981)[8] comparisons. The Brown-Forsythe test had better statistical power than other tests when the samples were from non-normal distributions, while it kept nominal Type I error rate. To our knowledge, the Brown-Forsythe test has not yet been applied to DNA methylation data.

Recently, two tests for the equality of variance (denoted as PO.AD and PO.SQ) were proposed in Phipson and Oshlack (2014)[10], which compared their two equal-variance tests with the F test and Bartlett's test using simulated data generated from a Bayesian hierarchical model, conditional distributions of which are normal distributions. Phipson and Oshlack (2014)[10] also evaluated the effect of outliers. However, they did not compare their tests with robust tests like the Brown-Forsythe test and did not investigate the effect of conditional non-normal distributions. Moreover, Phipson and Oshlack (2014)[10] only provided point estimates of performance measurements (e.g. type I error rate and power). The variances of these point estimates were not given, so we could not tell if the differences between the equal-variance tests observed in the paper are statistically significant or not.

In this article, we aimed to help researchers choose the right test of equal variance for DNA methylation data analysis. We compared Phipson and Oshlack's (2014)[10] two equal-variance tests with 5 commonly used equal-variance tests in the literature (classic F test, Bartlett's test, Levene's test, trimmed-mean-based Levene's test, and Brown-Forsythe test) via systematic simulation studies. We evaluated the effects of sample size, inequality of means, non-normal distribution, and outliers on the performances of the 7 equal-variance tests. We also evaluated if the differences among the performances of the 7 tests are statistically significant or not.

In addition to systematic simulation studies, we compared these 7 equal-variance tests by using two public available DNA methylation data sets GSE37020[6] and GSE20080[11] from Gene Expression Omnibus (GEO) (www.ncbi.nlm.nih.gov/geo), which was analyzed by Teschendorff and Widschwendter (2012)[6] to detect differentially variable DNA methylation markers.

## Materials and Methods

### Scientific question

The scientific question that we would like to address is whether the variances of two populations (e.g., diseased and non-diseased subjects) are the same based on samples drawn from the 2 populations. Specifically, let $\left\{x_{1},\cdots ,x_{m_{d}+m_{n}}\right\}$ be samples from two populations, where the population membership is indicated by the indicators $\left\{y_{1},\cdots ,y_{m_{d}+m_{n}}\right\}$, *m*_*d*_ and *m*_*n*_ are the number of diseased subjects and non-diseased subjects, respectively. *y*_*i*_ *= 1* indicates the *i*-th subject is a diseased subject; *y*_*i*_ *= 0* indicates the *i*-th subject is a non-diseased subject.

We would like to test the null hypothesis $H_{0}:\;\sigma_{d}^{2}=\sigma_{n}^{2}$ versus the alternative hypothesis $H_{a}:\;\sigma_{d}^{2}\neq \sigma_{n}^{2},$ where $\sigma_{d}^{2}$ and $\sigma_{n}^{2}$ are the variances of the diseased subjects and non-diseased subjects, respectively.

### The 7 statistical tests for testing equal variances

In this article, we would like to compare the performances of the 7 equal-variance tests: F test, Bartlett's test, Levene's test, trimmed-mean-based Levene's test, Brown Forsythe test, Phipson and Oshlack's (2014)[10] equal variance test based on absolute difference, and Phipson and Oshlack's equal variance test based on squared difference. We denoted the 7 tests by F, Bartlett, Levene, L.trim, BF, PO.AD, and PO.SQ, respectively.

The F test is based on the ratio of the variances of the 2 samples for two-group comparison. Bartlett's test was proposed to extend the F test for testing equal variance for more than 2 samples. Levene, L.trim, BF, PO.AD, and PO.SQ tests utilize the ideas of equal-mean tests (e.g., t-test or one-way ANOVA) and replace the original data *x*_*ik*_ in the test statistics by the transformed data *z*_*ik*_ = |*x*_*ik*_−*c*| or *z*_*ik*_ = (*x*_*ik*_−*c*)^2^, where the subscription *i* indicates subject, *k* indicates group, and *c* is a centrality measure, such as within-group mean or overall mean.

Specifically, Levene, L.trim, and BF tests replace *x*_*ik*_ by *z*_*ik*_ in one-way ANOVA's F test statistic; and PO.AD and PO.SQ tests replace *x*_*ik*_ by *z*_*ik*_ in the moderated t-test statistic [12].

The definitions of these 7 equal-variance tests are given in S1 File.

### Simulation studies

We considered 2 sets of simulation studies. One set is based on Ahn and Wang's (2013)[13] simulation studies. The other set is based on Phipson and Oshlack's (2014)[10] simulation studies.

Each of the 2 sets of simulation studies contained several scenarios. For each scenario, we generated 100 simulated data sets. For each simulated data set, we generated DNA methylation levels for 1000 CpG sites. The number of diseased subjects is set to be equal to the number of non-diseased subjects. To evaluate the effect of sample size, we considered 3 different sample sizes: 20 (small sample size), 50 (medium sample size) and 200 (large sample size) per group of subjects.

For each CpG site, we tested if the DNA methylation is differentially variable between diseased and non-diseased subjects using each of the 7 equal-variance tests. A test was claimed as significant if its p-value is <0.05. Two-sided tests were used. The R statistical software[14] was used to conduct the simulation studies.

### Simulation Study I

In Simulation Study I, we generated data sets based on Ahn and Wang's (2013)[13] simulation studies. Ahn and Wang (2013)[13] designed their simulation studies to evaluate the performance of their *joint* test that simultaneously tests for equal mean and equal variance. Following Ahn and Wang (2013)[13], we (1) generated DNA methylation levels from chi squared distributions and t distributions, in addition to normal distributions; and (2) considered the 2 scenarios of group means: (i) equal group means, and (ii) different group means.

Ahn and Wang’s (2013)[13] simulation studies did not evaluate the effect of outlier. We followed Phipson and Oshlack's (2014)[10] simulation studies to generate one outlier for each CpG site by replacing the DNA methylation level of one diseased subject by the maximum of the DNA methylation levels of all CpG sites.

The distribution settings for the scenarios in Simulation Study I are summarized in Table 1. Simulation study I had 3 (distributions) × 2 (scenarios of group means) × 2 (with or without outlier) × 3 (sample sizes) = 36 different comparisons.

**Table 1:** The distribution settings for the scenarios in Simulation Study I.

	chi squared distribution		t distribution		normal distribution	
Mean & variance	Non-D	D	Non-D	D	Non-D	D
**eqM & eqV (mean, var)**	$\chi_{2}^{2}$ (2, 4)	$\chi_{2}^{2}$ (2,4)	*t*_10_ (0, 1.25)	*t*_10_ (0, 1.25)	*N*(0,1) (0, 1)	*N*(0,1) (0, 1)
**diffM & eqV (mean, var)**	$\chi_{2}^{2}$ (2, 4)	$\chi_{1,\;0.5}^{2}$ (1.5, 4)	*t*_10_ (0, 1.25)	*t*_15,1.489_(1.57, 1.25)	*N*(0,1) (0, 1)	*N*(1.5,1) (1.5, 1)
**eqM & diffV (mean, var)**	$\chi_{2}^{2}$ (2, 4)	$\chi_{0.5,\;1.5}^{2}$ (2, 7)	*t*_10_ (0, 1.25)	*t*_10/3_ (0, 2.5)	*N*(0,1) (0, 1)	*N*(0,2) (0, 2)
**diffM & diffV (mean, var)**	$\chi_{2}^{2}$ (2, 4)	$\chi_{4}^{2}$ (4, 8)	*t*_10_ (0, 1.25)	*t*_6,2.393_ (2.75, 2.5)	*N*(0,1) (0, 1)	*N*(1.5,2) (1.5, 2)

eqM: equal-mean; eqV: equal-variance; diffM: different-mean; diffV: different-variance; D: diseased; Non-D: non-diseased;*N(a*,*b)*: normal distribution with mean *a* and variance *b*; *t*_*c*_: t-distribution with degrees of freedom *c*; *t*_*d*,*e*_: non-central t-distribution with degrees of freedom *d* and non-centrality parameter *e*; $\chi_{f}^{2}$: chi squared distribution with degrees of freedom *f*; $\chi_{g,h}^{2}$: non-central chi squared distribution with degrees of freedom *g* and non-centrality parameter *h*.

### Simulation Study II

Simulation study II was based on Phipson and Oshlack's (2014)[10] simulation studies.

Phipson and Oshlack (2014)[10] generated DNA methylation levels from Bayesian hierarchical models to allow correlations among CpG sites. M-values [15], i.e., logistically transformed Illumina's β-values, were used to measure DNA methylation levels. For a CpG site, given its variance the M-values of DNA methylation levels were generated from normal distributions. Diseased subjects and non-diseased subjects have mean M-values 2 and -2, respectively. The variances themselves are random variables from a scaled inverse chi squared distribution scale-inv-$\chi^{2}\left(d_{0},s_{0}^{2}\right)$ with the degrees of freedom *d*_0_ and the scale factor $s_{0}^{2}$.

In Simulation Study II, we first generated the M-values of DNA methylation levels from Bayesian hierarchical models with conditional normal distribution to compare 7 equal-variance tests. We then evaluated the effect of non-normal distribution, by generating the M-values of DNA methylation levels from Bayesian hierarchical models with conditional chi squared distribution, the degrees of freedom of which were generated from the scaled inverse chi squared distribution scale-inv-$\chi^{2}\left(d_{0},s_{0}^{2}\right)$.

Following Phipson and Oshlack (2014)[10], to evaluate type I error rate, we set the degrees of freedom *d*_0_ = 20 and the scaling factor $s_{0}^{2}=0.64$ for both non-diseased and diseased subjects. To evaluate the power of the tests, we set the scaling factor as $s_{0}^{2}=0.64$ for non-diseased subjects and $s_{0}^{2}=1.5$ for diseased subjects. The degrees of freedom are set to be *d*_0_ = 20 for both non-diseased and diseased subjects.

We next evaluated the effect of outliers on the performances of the 7 tests by replacing the M-value of one case subject with the maximum M-value of all CpG sites for all subjects.

Simulation study II had 2 (distributions) × 2 (with or without outlier) × 3 (sample sizes) = 12 different comparisons.

### GSE37020 and GSE20080 data sets

To evaluate the performance of the 7 equal-variance tests for real data sets, we used two data sets (GSE37020 and GSE20080) downloaded from the public repository: Gene Expression Omnibus (GEO, www.ncbi.nlm.nih.gov/geo). Both data sets contain DNA methylation (DNAm) profiles of 27,578 CpG sites measured from liquid based cytology (LBC) cervical smear samples by IlluminaHumanMethylation27 platform.

GSE37020 contains a total of 48 samples, 24 of which had normal histology and 24 of which were cervical intraepithelial neoplasia of grade 2 or higher (CIN2+). All of them are human papillomavirus (HPV) positive. Normal and CIN2+ samples are age-matched. GSE20080 also contains 48 samples. A total of 30 samples (HPV positive: 11 samples and HPV negative: 19 samples) have normal cytology. The other 18 samples (all HPV positive) are with CIN2+. Moreover, normal and CIN2+ samples were age-matched. For both of the data sets, we excluded some CpG sites residing on SNPs or with missing values. Quantile plots and principal component analysis did not show obvious patterns (c.f. Figs A and B in S1 File). After data QC, GSE37020 has 23,066 CpG sites and 48 samples and GSE20080 has 23,255 CpG sites and 48 samples. There were 22,859 CpG sites appearing in both cleaned data sets. We used these 22,859 CpG sites in our real data analysis.

We used GSE37020 as the discovery set to detect CpG sites differentially variable between normal cytology samples and CIN2+ samples. To control multiple testing, we applied the Benjamini and Hochberg method[16] to adjust p-values so that the false discovery rate (FDR) is controlled at the level of 0.05. Specifically, a CpG site was claimed significant if its FDR-adjusted p-value is <0.05. We then validated these differentially variable CpG sites by using the GSE20080 data set. If an equal-variance test for a given CpG site had FDR-adjusted p-value < 0.05 in the analysis of GSE37020 and had un-adjusted p-value < 0.05 in the analysis of GSE20080, we then claimed that the significance of the test in GSE37020 is validated in GSE20080.

### Measure of performance

For each simulated data set, we measured the performance of an equal-variance test by the estimated type I error rate and power. The estimated type I error rate is the proportion of significant tests detected by the equal-variance test among the 1000 CpG sites in a simulated data set generated from the null hypothesis (i.e., CpG sites are non-differentially variable). Estimated power is the proportion of significant tests detected by the equal-variance test among the 1000 CpG sites in a simulated data set generated from the alternative hypothesis (i.e., CpG sites are differentially variable).

For the real data analysis, among the significant CpG sites in testing for equality of variance based on GSE37020 we calculated the numbers (proportions) of CpG sites that validated in the test data set GSE20080. We also identified CpG sites having outliers (i.e., DNA methylation level lies more than 1.5 times the interquartile range below the first quartile or above the third quartile of DNA methylation levels across the arrays) either in the case group or control group, or both for each of the 7 equal-variance tests. We then calculate the numbers (proportions) of validated significant CpG sites with outliers based on GSE20080.

## Results

### Results of simulation studies

By using simulation studies, we evaluated the effects of sample size, inequality of means, non-normal distribution, and outliers on the performances of the 7 equal-variance tests.

The parallel boxplots of the estimated type I error rates/powers for all 48 scenarios in the 2 simulation studies are shown in Figs C to R in S1 File.

These figures showed that as sample size increases, the performances of the 7 tests improve. That is, the estimated type I error rates are closer to the nominal value 0.05 and the estimated power is closer to the maximum value 1 as the sample size increases from 20 subjects per group to 200 subjects per group. The performances for sample size 20 or 50 subjects per group were similar to each other and were of limited power (<0.5) for many scenarios where data were generated from non-normal distributions. The performance for data sets with 200 subjects per group were much better than those for data sets with 20 or 50 subjects per group and had adequate power (>0.8) for most of scenarios. In addition, the inequality of means has no effect on all 7 tests.

Both non-normality and outliers had effects on the performances of the 7 equal-variance tests. Consistent with what is reported in the literature, F and Bartlett's tests were much more sensitive (i.e., having large type I error rates) to non-normality and outliers than the other 5 tests.

From Figs C to R in S1 File, we also had the following observations. Firstly, the F test and Bartlett's test had almost identical performances and performed best when data were generated from (conditional) normal distributions (c.f. Figs M, N, and Q in S1 File). However, the F test and Bartlett's test had type I error rates higher than the nominal value 0.05 when data were generated from a non-normal distribution with/without outliers or from a normal distribution with outliers.

Secondly, the PO.AD test and Levene's test tend to have type I error rates higher than the nominal value 0.05 in almost all scenarios, even when the DNA methylation levels were generated from a normal distribution as in Simulation I or conditional normal distribution as in Simulation II (c.f. Figs M, N, and Q in S1 File).

However, the PO.AD test and Levene's test had the highest power in almost all scenarios where the two tests kept the nominal type I error rate (0.05).

Finally, the L.trim, BF and PO.SQ tests had type I error rates close to or smaller than 0.05 and had relatively good power in almost all scenarios.

**Fig 1:**
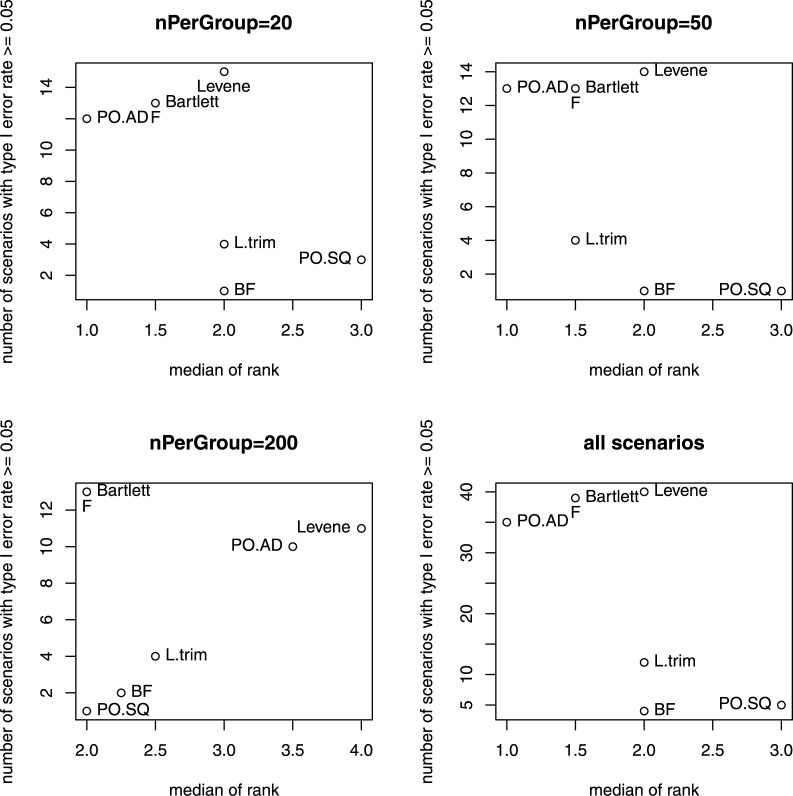
Plots of *n*_*reject*_ versus *m*, where *n*_*reject*_ is the number of scenarios where an equal-variance test rejected the null hypothesis *H*_*0*_ that the type I error rates is ≤ 0.05 and *m* is the median of the ranks of powers. For ranks with ties, average ranks were used. The upper-left, upper-right, bottom-left panels are the plots where *n*_*reject*_ and *m* were obtained based on scenarios with sample size 20, 50, or 200 subjects per group, respectively. The bottom-right panel is the plot where *n*_*reject*_ and *m* were obtained based on all 48 scenarios.

Table A in S1 File shows the ranks of the 7 equal-variance tests in terms of power for each scenario in the 2 simulation studies. Specifically, for each scenario, we first tested for each equal-variance test the null hypothesis H_0_ that the type I error rate of the equal-variance test is equal to or smaller than the nominal value 0.05 based on the 100 simulated data sets. For those equal-variance tests that did not reject the null hypothesis H_0_, we then ranked them in terms of power. For those equal-variance tests that rejected H_0_, we set the rank as missing values. We further obtained the median *m* of the ranks for each of the 7 equal-variance tests and the corresponding number *n*_*reject*_ of scenarios that rejected H_0_. For ranks with ties, average ranks were used. Fig 1 illustrates four plots of *n*_*reject*_ versus *m*. The upper-left, upper-right, and bottom-left panels are the plots where *n*_*reject*_ and *m* were obtained based on scenarios with sample size 20, 50, or 200 subjects per group, respectively. The bottom-right panel is the plot where *n*_*reject*_ and *m* were obtained based on all 48 scenarios.

Fig 1 and Table A in S1 File confirmed several observations we obtained based on the parallel boxplots (c.f. Figs C to R in S1 File):

(1) F test, Bartlett's test, Levene's test and PO.AD test had type I error rates higher than the nominal value (0.05) in almost all scenarios, while the other 3 tests kept nominal type I error rates for almost all scenarios; (2) F test and Bartlett's test had very similar performance and performed best under normality assumption, while the 2 tests had type I error rates higher than the nominal value (0.05) when the normality assumption is violated; (3) Levene's test and PO.AD test had better power than the other 5 tests for almost all scenarios where the 2 tests kept the nominal type I error rates; (4) Among the L.trim, BF, and PO.SQ tests, BF test performed best, followed by L.trim and PO.SQ test; and (5) the power improved a lot by increasing sample size from 20/50 subjects per group to 200 subjects per group. In addition, we observed that the ranks of the 7 tests did not change much as the sample size increases.

### Results from GSE37020 and GSE20080

For the real data set GSE37020, the numbers of significant CpG sites (i.e., CpG sites with FDR-adjusted p-value < 0.05) obtained by the 7 equal-variance tests are 2318 (F), 2315 (Bartlett), 235 (Levene), 15 (L.trim), 7 (BF), 130 (PO.AD), and 0 (PO.SQ), respectively. The numbers of significant CpG sites detected by F test and Bartlett test are much larger than those detected by other tests. No significant CpG sites were detected by PO.SQ test.

The numbers/proportions of significant CpG sites validated by GSE20080 are 1154/49.8% (F), 1164/50.3% (Bartlett), 183/77.9% (Levene), 9/60.0% (L.trim), 3/42.9% (BF), and 91/70% (PO.AD), respectively (c.f. Table 2). That is, all the 6 tests had large proportion of significant CpG sites validated by the testing set GSE20080. However, overall the robust equal-variance tests had larger proportions of validated significant CpG sites than F or Bartlett test.

**Table 2:** Number of significant CpG sites (FDR-adjusted p-value < 0.05) in testing for equality of variance based on GSE37020, and the numbers and proportions of significant CpG sites validated via GSE20080 (unadjusted p-value < 0.05). Total number of CpG sites is 22859.

	GSE37020	GSE20080	
test	nCpG (p.adj<0.05)	nCpG.validated (pval<0.05)	Proportion*
F	2318	1154	49.8%
Bartlett	2315	1164	50.3%
Levene	235	183	77.9%
L.trim	15	9	60.0%
BF	7	3	42.9%
PO.AD	130	91	70%
PO.SQ	0	0	-

*: proportion = nCpG (p.adj<0.05) / nCpG.validated (pval<0.05).

Since F test and Bartlett’s test are sensitive to outliers, we checked the number/proportion of significant CpG sites containing outliers detected based on GSE37020. The numbers/proportions are 1503/64.8% (F), 1501/64.8% (Bartlett), 70/29.8% (Levene), 2/13.3% (L.trim), 2/28.6% (BF), and 64/49.2% (PO.AD), respectively (c.f. second column of Table 3). For F test and Bartlett’s test, more than 60% significant CpG sites contain outliers. For robust tests (e.g., Levene, L.trim, and BF), the proportions are relatively small (<30%).

**Table 3:** Number/proportion of significant CpG sites that contain outliers detected in GSE37020, and the number/proportion of these CpG sites that also contains outliers detected via GSE20080.

	GSE37020	validation
test	nOutlier/pOutlier*	nOutlier/pOutlier**
F	1503/64.8%	495/32.9%
Bartlett	1501/64.8%	497/33.1%
Levene	70/29.8%	34/48.6%
Trim.mean	2/13.3%	0/0%
BF	2/28.6%	0/0%
PO.AD	64/49.2%	31/48.4%
PO.SQ	0/-	0/-

*: Number/proportion of significant CpG sites containing outliers detected in GSE37020;

**: Number/proportion of significant CpG sites that contain outliers detected in both GSE37020 and GSE 20080.

We then checked if the significant CpG sites containing outliers in GSE37020 would still contain outliers in GSE20080. The number/proportion of such CpG sites are 495/32.9% (F), 497/33.1% (Bartlett), 34/48.6% (Levene), 0/0% (L.trim), 0/0% (BF), and 31/48.4% (PO.AD), respectively (c.f. third column of Table 3).

**Fig 2:**
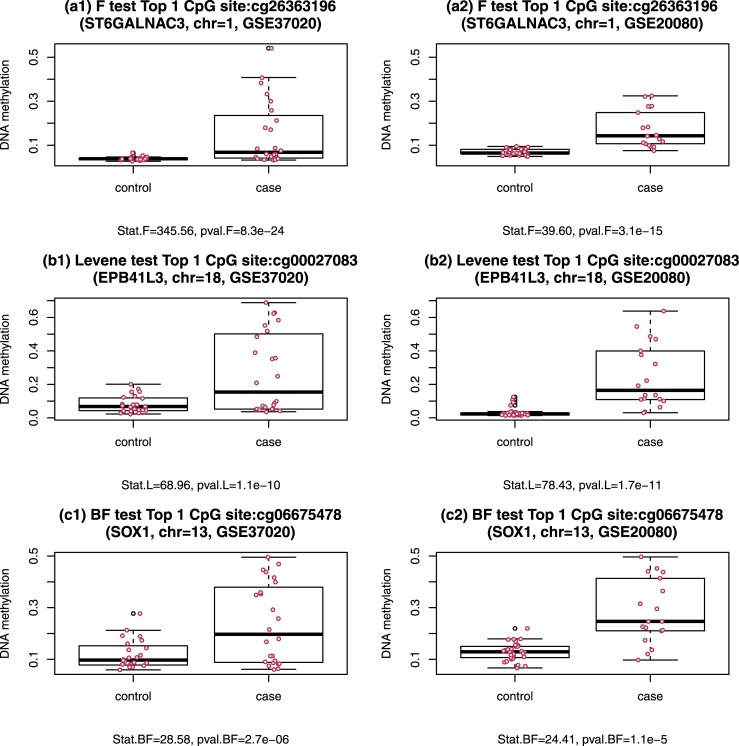
Paired parallel boxplots of DNA methylation level versus case-control status for the 4 unique top 1 CpG sites obtained by the 7 equal-variance tests based on GSE37020. The red dots indicate subjects. 2a1, 2b1, 2c1 are for cg26363196 (F), cg00027083 (Levene), and cg06675478 (BF), respectively, based on GSE37020; 2a2 2b2, 2c2 are for cg26363196 (F), cg00027083 (Levene), and cg06675478 (BF), respectively, based on GSE20080.

We next checked the parallel boxplots of DNA methylation level versus case-control status for the top 1 CpG site (i.e., having the smallest p-value for testing equal variance) obtained by each of the 7 tests based on GSE37020. The top 1 CpG sites detected by the 7 equal-variance tests are cg26363196 (F, Bartlett, PO.AD, and PO.SQ), cg00027083 (Levene and L.trim), and cg06675478 (BF), respectively. All these top CpG sites were validated in GSE20080. Fig 2 shows the boxplots of these 3 unique top 1 CpG sites. We found that all these 3 top CpG sites contain at least one outlier in either GSE37020, or GSE20080.

## Discussion

Recently, two new tests of equality of variance were proposed in the literature of DNA methylation data analysis by Phipson and Oshlack's tests[10]. However, their performance has not been compared with existing tests that are robust to the violation of normality assumption, such as the classical Levene's test, trimmed-mean-based Levene's test, and Brown-Forsythe test.

In this article, we systematically compared the performance of the 2 new equal-variance tests with the F test, Bartlett's test, Levene's test, trimmed-mean-based Levene's test, and Brown-Forsythe test via two simulation studies and one real-data analysis.

The simulation studies showed that (1) the BF test and L.trim had better performance than the other 5 tests in terms of having high power while keeping the nominal type I error rate; (2) the PO.AD test tends to have a type I error rates higher than the nominal value 0.05 for a majority of simulation scenarios, while the PO.SQ test could keep the nominal type I error rate of 0.05 for almost all simulation scenarios; and (3) for almost all of the scenarios where PO.AD test kept the nominal type I error rate, PO.AD test had the highest power. The PO.SQ test was less powerful than the F, Bartlett's, L.trim, and/or BF tests for almost all of scenarios where the PO.SQ test kept the nominal type I error rate.

By examining the definitions of the PO.AD and PO.SQ tests (c.f. Section A.2 in S1 File), we found that the outcomes *z*_i_* (*z*_i_**), *i = 1*, *…*, *m*_*c*_ *+m*_*n*_, in the linear regressions (c.f. Formula A1 in S1 File) are conditionally correlated and not conditionally normally distributed. However, Phipson and Oshlack's (2014) tests require these two assumptions: given a CpG site *i*, *z*_i_* (*z*_i_**) *i = 1*,*…*, *m*_*c*_ *+m*_*n*_ are independent and normally distributed. Hence, cautions are needed when applying for the PO.AD or PO.SQ tests to test for equality of variance.

Based on the above observations, BF or L.trim test for equality of variance has relatively high power while keeping the nominal type I error rate for almost all of the 48 simulation scenarios. It is also a surprise that Levene's test had type I error rates higher than the nominal value 0.05 for a majority of the scenarios, even for the scenarios where data were generated from a normal distribution.

In our simulation studies, the powers of the 7 equal-variance tests for data generated from chi squared distributions were low. Data transformation might be helpful.

In our Simulation Study I, DNA methylation levels for different CpG sites were generated independently, while different CpG sites generated in our Simulation Study II were marginally correlated, but conditionally independent, by using Bayesian hierarchical models. Compared to real DNA methylation data, our simulation studies are simple and could not cover all scenarios encountered in real DNA methylation data analysis. However, our simulation studies do provide useful information about the performance of the 7 equal-variance tests.

We noticed that in DNA methylation data analyses shown in literature, F/Bartlett's test was generally used to detect differential variable DNA methylation markers. It is well known in statistics that F/Bartlett's test is very sensitive to outliers and non-normality. Our simulation studies and real data analysis confirmed this. However, outliers might be biologically important as pointed by Teschendorff and Widschwendter (2012)[6]. The real data analysis in this article also showed that a number (30%–50%) of significant CpG sites with outliers detected in the discovery set GSE37020 also contained outliers in the testing set GSE20080. Our real data analysis also agreed with Teschendorff and Widschwendter’s (2012) observation that changes in DNA methylation for differentially variable CpG sites are heterogeneous and stochastic as shown in the parallel boxplots for CpG cg26363196 in Fig 2.

We also noticed in the real data analyses that some outliers might be due to technical effects. For example, more than 60% of the 1503 significant CpG sites that were detected by F test and contain outliers based on the discovery set GSE37020 did not contain outliers in the testing set GSE20080. For a real data set, there might be no independent data sets available to check if the outliers in the significant CpG sites are due to technical effects or not. In this case, one might do bootstrapping and apply several equal-variance tests (such as F test, Bartlett’s test, Levene’s test, L.trim, BF, and PO.AD test). It would be assuring if a CpG site is consistently claimed as a differentially variable CpG site in different bootstrapping samples and by using different equal-variance tests.

The score test proposed by Ahn and Wang (2013)[13] aimed to simultaneously test for equality of mean and equality of variance for improving power of identifying methylation markers related to disease, rather than to test for equality of variance alone. Hence, we did not compare their score test in this article.

Finally, we would like to emphasize that although the idea of differential variability was first discussed in the context of cancer [4], the added value of differential variability over differential methylation seems to be clearest in the context of normal to normal comparisons, as demonstrated by Teschendorff et al. (2012) [17], in which they showed that analysis of epigenetic variability in prospectively collected normal cells can predict the risk of future morphological transformation.

## Supporting Information

S1 File Supplementary Documents. The definitions of the 7 equal-variance tests, QC plots for the 2 GEO data sets (GSE37020 and GSE20080), parallel boxplots for simulation studies, and the table of ranks in terms of power for simulation studies.
(PDF)
