# Supplementary material for: A Comparative Study of Tests for Homogeneity of Variances with Application to DNA Methylation Data
Source: PLoS One. 2015 Dec 18;10(12):e0145295. doi: 10.1371/journal.pone.0145295 (PMC4684215; doi:10.1371/journal.pone.0145295)
Supplement: S1 File — The definitions of the 7 equal-variance tests, QC plots for the 2 GEO data sets (GSE37020 and GSE20080), parallel boxplots for simulation studies, and the table of ranks in terms of power for simulation studies. (PDF) [file pone.0145295.s001.pdf]

Supplementary Documents for "A comparative study of tests for homogeneity of variances with application to DNA methylation data"

Xuan Li[1], Weiliang Qiu[2], Jarret Morrow[2], Dawn L. DeMeo[2], Scott T. Weiss[2], Yuejiao Fu[1], Xiaogang Wang[1]

[1] Department of Mathematics and Statistics,

York University,

4700 Keele Street, Toronto, ON, M3J1P3, Canada

[2] Channing Division of Network Medicine, Brigham and Women's Hospital, Harvard Medical School, 181 Longwood Avenue, Boston, 02115, USA

A Test statistics

A.1 Phipson and Oshlack's tests (PO.AD and PO.SQ tests)

Phipson and Oshlack (2014)[1] proposed 2 equal-variance tests based on the following 2 linear regressions:

$$\begin{aligned} z_i^* &= \beta_0 + \beta_1 y_i + \epsilon_i, \\ z_i^{**} &= \gamma_0 + \gamma_1 y_i + \xi_i, \\ i &= 1, \ldots, m_c + m_n, \end{aligned} \tag{A1}$$

where

$$\begin{aligned} z_i^* &= c_i^* \left| x_i - g\left(y_i\right) \right|, \\ z_i^{**} &= c_i^* \left[x_i - g\left(y_i\right) \right]^2, \end{aligned}$$

and $g(y_i)$ is the group mean for cases if $y_i = 1$ or the group mean for controls if $y_i = 0$.

The value of c_i^* is

$$c_i^* = \begin{cases} \sqrt{\frac{m_c}{m_c - 1}} & \text{if } y_i = 1 \text{ (i.e., case)}, \\ \sqrt{\frac{m_n}{m_n - 1}} & \text{if } y_i = 0 \text{ (i.e., control)}, \end{cases}$$

Phipson and Oshlack (2014)[1] mentioned that to test for equal-variance between cases and controls is equivalent to test if the slope β_1 (γ_1) is equal to zero.

Phipson and Oshlack (2014)[1] applied moderated t-test (Smyth, 2004)[2] to borrow information across CpG sites to improve the test of the null hypothesis that the slope $\beta_1 = 0$ ($\gamma_1 = 0$) for a given CpG site.

Note that linear regressions in Formula (A1) assume ϵ_i (ξ_i) $i = 1, \ldots, m_c + m_n$ are independent and normally distributed. Or equivalently, z_i^* (z_i^{**}) $i = 1, \ldots, m_c + m_n$ are independent and normally distributed. However, each z_i^* (z_i^{**}) contains group means $g(y_i)$. Hence, z_i^* (z_i^{**}) $i = 1, \ldots, m_c + m_n$ are correlated within case and control groups. Moreover, the distribution of $z_i^* = c_i^* |x_i - g(y_i)|$ ($z_i^{**} = c_i^* [x_i - g(y_i)]^2$) could not be normally distributed. Hence, cautions are needed when applying for the PO.AD or PO.SQ tests to test for equality of variance.

A.2 F test

The F test statistic is asymptotically F distributed under the null hypothesis (i.e. equal variance)

$$F = \frac{S_1^2}{S_0^2} \to F_{n_1 - 1, n_0 - 1},$$

where

$$
\begin{aligned}
S_k^2 =& \frac{1}{(n_k - 1)} \sum_{i=1}^{n_k} (x_{k,i} - \bar{x}_k)^2 \\
S_p^2 =& \frac{1}{(N-2)} \sum_{k=1}^{2} (n_k - 1) S_k^2 \\
\bar{x}_k =& \frac{1}{n_k} \sum_{i=1}^{n_k} x_{k,i}, k = 0, 1,
\end{aligned}
$$

n_0 and n_1 are the sample sizes for controls and cases, respectively, and $N = n_0 + n_1$.

A.3 Bartlett's test

For 2 sample comparison ($K = 2$), Bartlett's test statistic is (`http://en.wikipedia.org/wiki/Bartlett's_test`)

$$
X^2 = \frac{(n_1 + n_0 - 2) \log\left(S_p^2\right) - \sum_{k=1}^{2} (n_k - 1) \log\left(S_k^2\right)}{1 + \frac{1}{3}\left(\sum_{k=1}^{2}\left(\frac{1}{(n_k - 1)}\right) - \frac{1}{(n_1 + n_0 - 2)}\right)} \to \chi_1^2,
$$

The numerator of the Bartlett's test for 2 sample comparison is

$$
\begin{aligned}
numer =& (n_1 + n_0 - 2) \log\left(S_p^2\right) - \left[(n_1 - 1) \log\left(S_1^2\right) + (n_0 - 1) \log\left(S_0^2\right)\right] \\
=& \log\left\{\frac{\left[(S_p)^2\right]^{n_1 + n_0 - 2}}{\left[(S_1)^2\right]^{n_1 - 1}\left[(S_0)^2\right]^{n_0 - 1}}\right\} \\
=& \log\left\{\left[\frac{(S_p)^2}{(S_1)^2}\right]^{n_1 - 1}\left[\frac{(S_p)^2}{(S_0)^2}\right]^{n_0 - 1}\right\} \\
=& \log\left\{\left[\frac{1}{(n_1 + n_0 - 2)}\left[(n_1 - 1) + (n_0 - 1)\frac{S_0^2}{S_1^2}\right]\right]^{n_1 - 1}\left[\frac{1}{(n_1 + n_0 - 2)}\left[(n_0 - 1) + (n_1 - 1)\frac{S_1^2}{S_0^2}\right]\right]^{n_0 - 1}\right\} \\
=& \log\left\{\left[\frac{1}{(n_1 + n_0 - 2)}\left[(n_1 - 1) + (n_0 - 1)\frac{1}{F}\right]\right]^{n_1 - 1}\left[\frac{1}{(n_1 + n_0 - 2)}\left[(n_0 - 1) + (n_1 - 1) F\right]\right]^{n_0 - 1}\right\},
\end{aligned}
$$

where

$$
F = \frac{S_1^2}{S_0^2}
$$

is the F test statistic.

Hence, Bartlett's test for 2 sample comparison is equivalent to F test, but both small sample size performance and large sample size performance will have slight difference due to different asymptotic distributions used.

A.4 Levene's test

Levene's test statistic for 2 sample case is defined as

$$W = \frac{(n-2)\left[n_1(\bar{w}_1-\bar{w})^2 + n_0(\bar{w}_0-\bar{w})^2\right]}{\sum_{i=1}^{n_1}(w_{1i}-\bar{w}_1)^2 + \sum_{j=1}^{n_0}(w_{0j}-\bar{w}_0)^2},$$

where

$$\begin{aligned}
w_{1i} &= |x_{1i} - \bar{x}_1|, \quad \bar{x}_1 = \frac{1}{n_1}\sum_{i=1}^{n_1} x_{1i}, \\
w_{0j} &= |x_{0j} - \bar{x}_0|, \quad \bar{x}_0 = \frac{1}{n_0}\sum_{j=1}^{n_0} x_{0i}, \\
\bar{w}_1 &= \frac{1}{n_1}\sum_{i=1}^{n_1} w_{1i}, \\
\bar{w}_0 &= \frac{1}{n_0}\sum_{j=1}^{n_0} w_{1j}, \\
\bar{w} &= \frac{1}{n}\left[\sum_{i=1}^{n_1} w_{1i} + \sum_{j=1}^{n_0} w_{0j}\right].
\end{aligned}$$

A.5 Trimmed-mean-based Levene's test

Trimmed-mean-based Levene's test for 2 sample comparison has the same format as Levene's test. The only difference is in the definition of w_{1i} and w_{0j}:

$$\begin{aligned}
w_{1i} &= |x_{1i} - trimmean(x_1)|, \\
w_{0j} &= |x_{0j} - trimmean(x_0)|,
\end{aligned}$$

where $trimmean(x_)$ and $trimmean(x_0)$ are within-group trimmed means for cases and controls, respectively.

A.6 Brown and Forsythe's test

Brown-Forsythe test statistic for 2 sample comparison has the same format as Levene's test. The only difference is in the definition of w_{1i} and w_{0j}:

$$w_{1i} = |x_{1i} - median(x_1)|\,,$$

$$w_{0j} = |x_{0j} - median(x_0)|\,.$$

B QC plots for GSE30760 and GSE20080

In real data analysis, we drew the plot of quantiles across arrays for GSE37020 and GSE20080, respectively, after data cleaning. No obvious patterns were found. (c.f. the left and right panels of the online Supplementary Figure A).

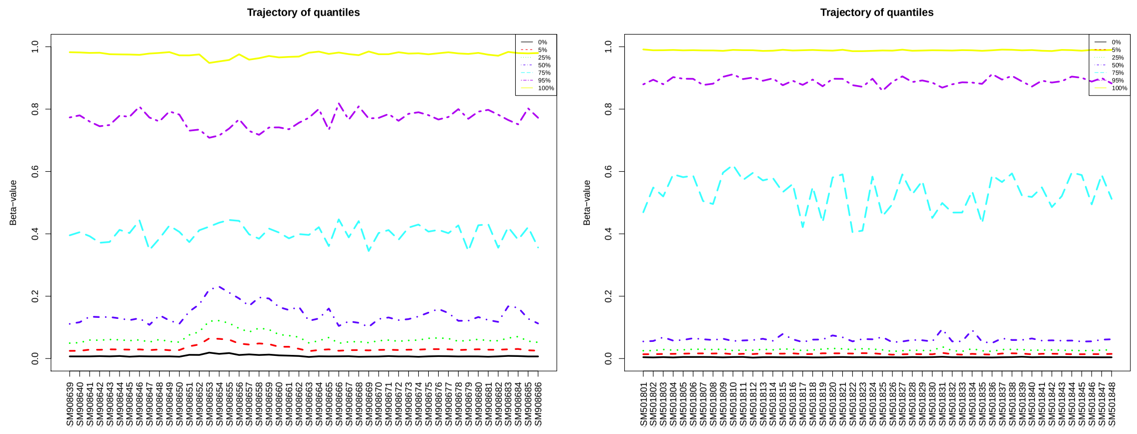

Figure A: The plot of quantiles across arrays. Left panel was for GSE37020; right panel was for GSE20080.

We also drew the scatter plot of the first principal component (PC1) versus the second principal component (PC2) for GSE37020 and GSE20080, respectively, after data cleaning.

No obvious patterns were found. (c.f. the left and right panels of the online Supplementary Figure B).

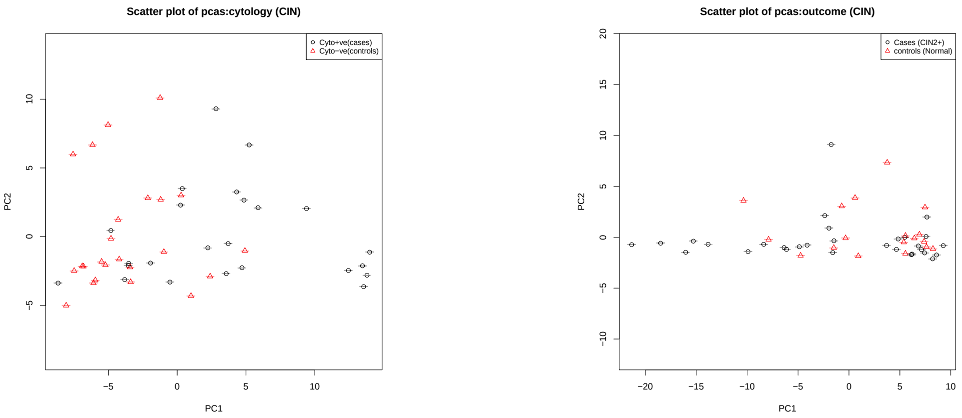

Figure B: The plot of the first principal component (PC1) versus the second principal component (PC2) for DNA methylation data. Left panel was for GSE37020; right panel was for GSE20080.

C Parallel boxplots for simulation studies

C.1 Data generated from (conditional) chi squared distributions

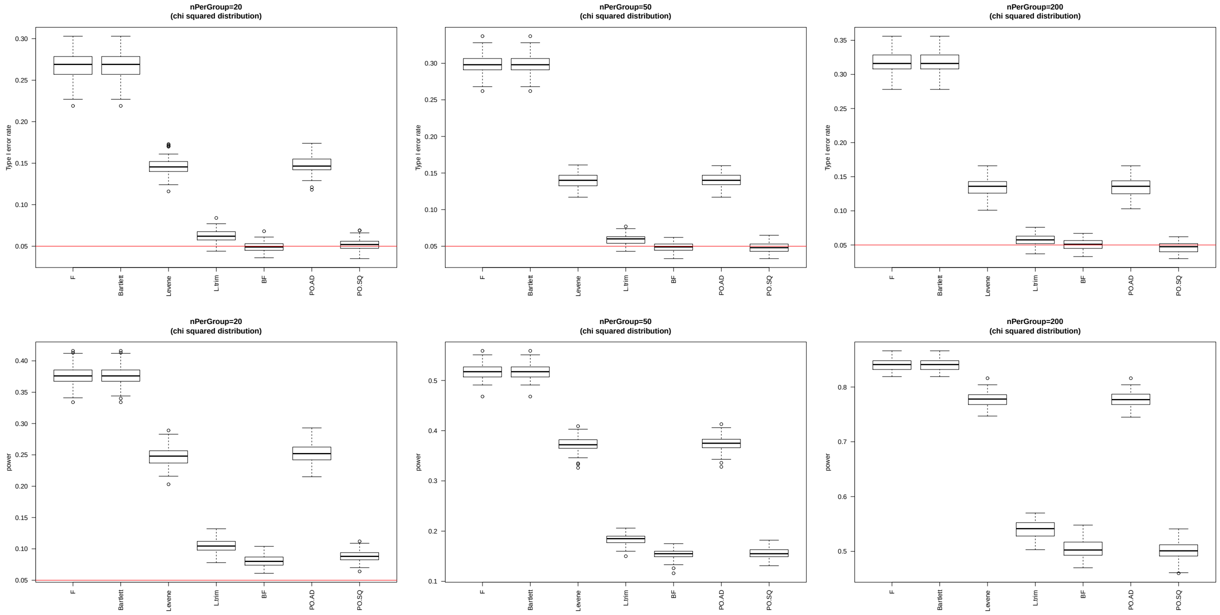

Figure C: Parallel boxplots for scenarios where data were generated from chi squared distributions with equal means. Upper panel: Type I error rates (The red horizontal line indicates Type I error rate = 0.05); Lower panel: powers (The red horizontal line indicates power = 0.05). Left panel: 20 subjects per group; middle panel: 50 subjects per group; right panel: 200 subjects per group.

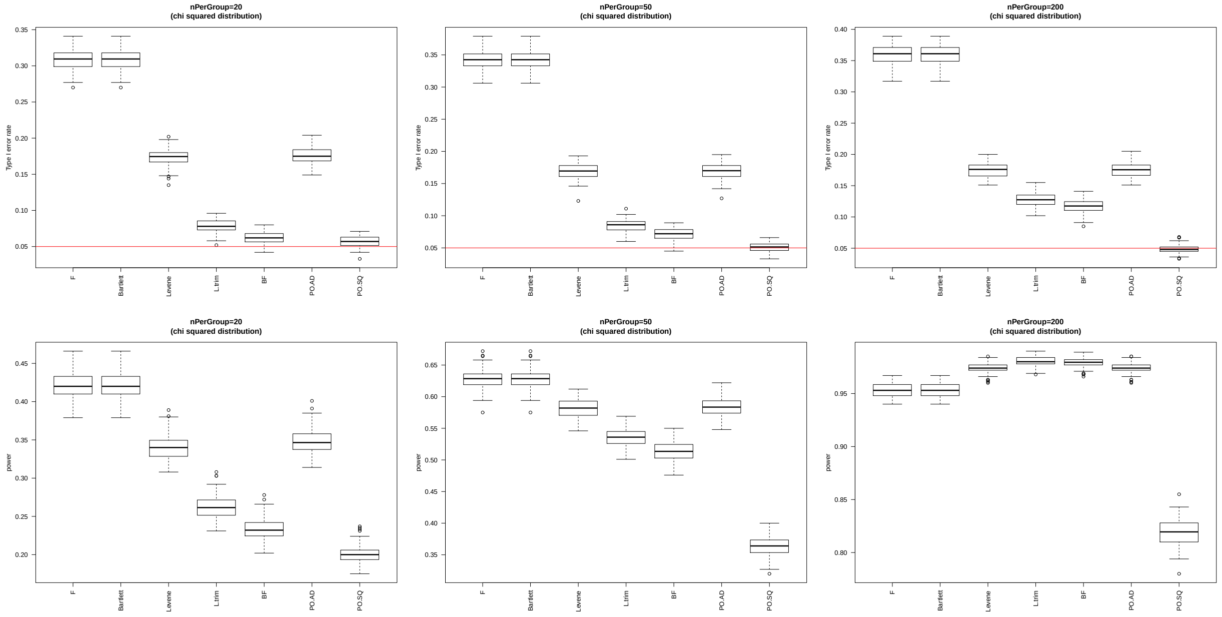

Figure D: Parallel boxplots for scenarios where data were generated from chi squared distributions with different means. Upper panel: Type I error rates (The red horizontal line indicates Type I error rate = 0.05); Lower panel: powers (The red horizontal line indicates power = 0.05). Left panel: 20 subjects per group; middle panel: 50 subjects per group; right panel: 200 subjects per group.

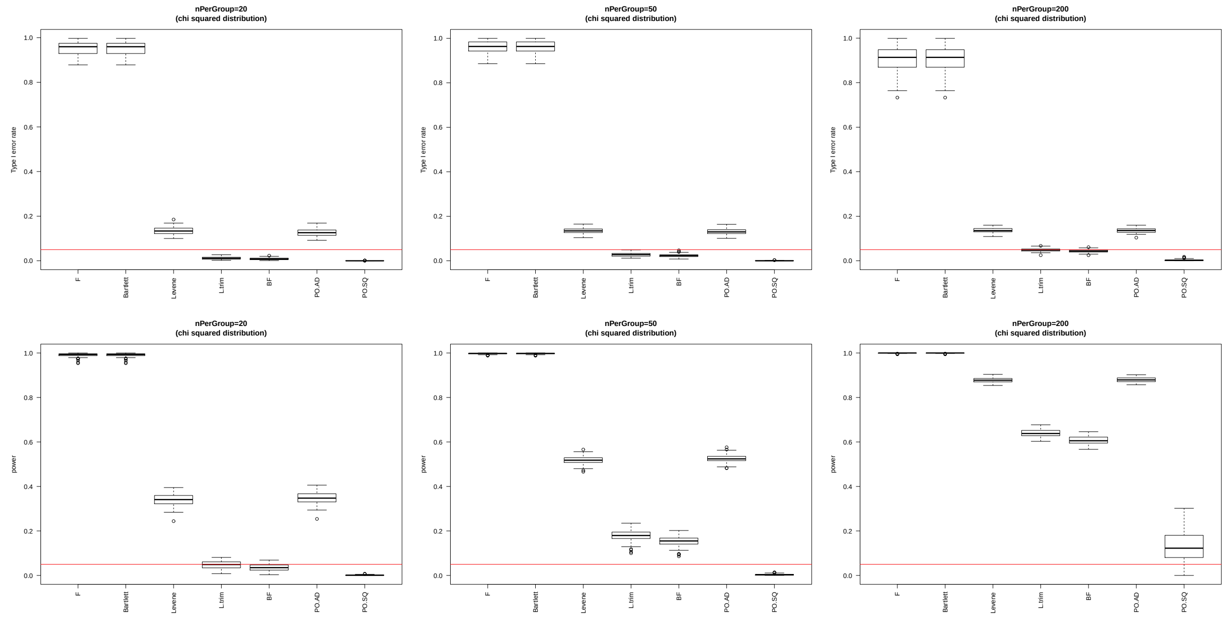

Figure E: Parallel boxplots for scenarios where data were generated from chi squared distributions with equal means and outlier. Upper panel: Type I error rates (The red horizontal line indicates Type I error rate = 0.05); Lower panel: powers (The red horizontal line indicates power = 0.05). Left panel: 20 subjects per group; middle panel: 50 subjects per group; right panel: 200 subjects per group.

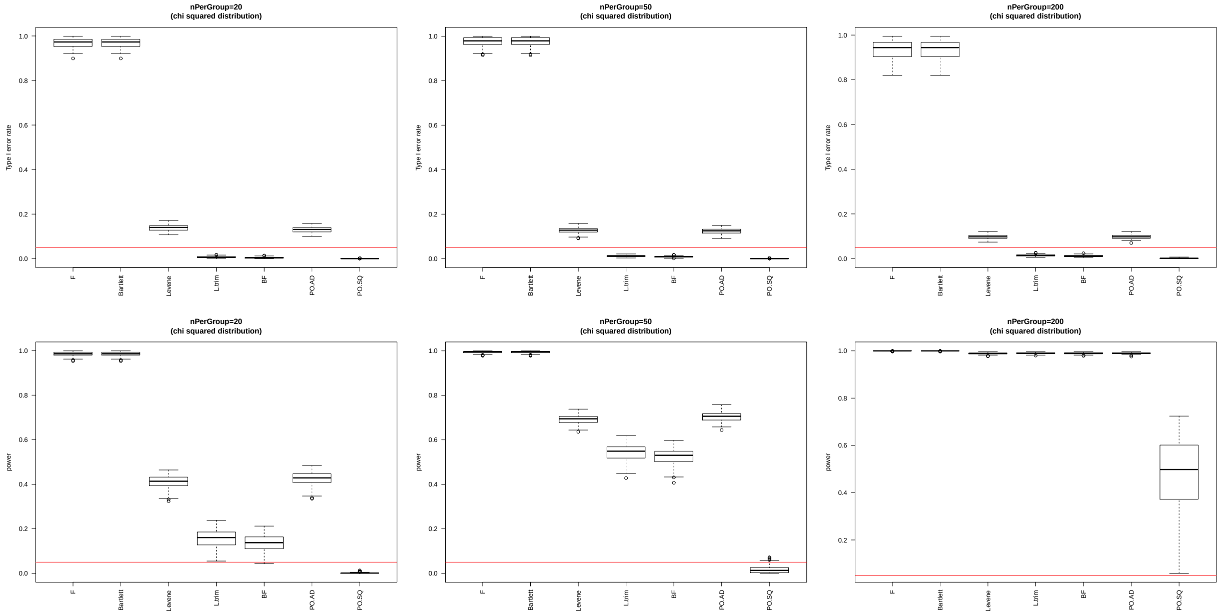

Figure F: Parallel boxplots for scenarios where data were generated from chi squared distributions with different means and outlier. Upper panel: Type I error rates (The red horizontal line indicates Type I error rate = 0.05); Lower panel: powers (The red horizontal line indicates power = 0.05). Left panel: 20 subjects per group; middle panel: 50 subjects per group; right panel: 200 subjects per group.

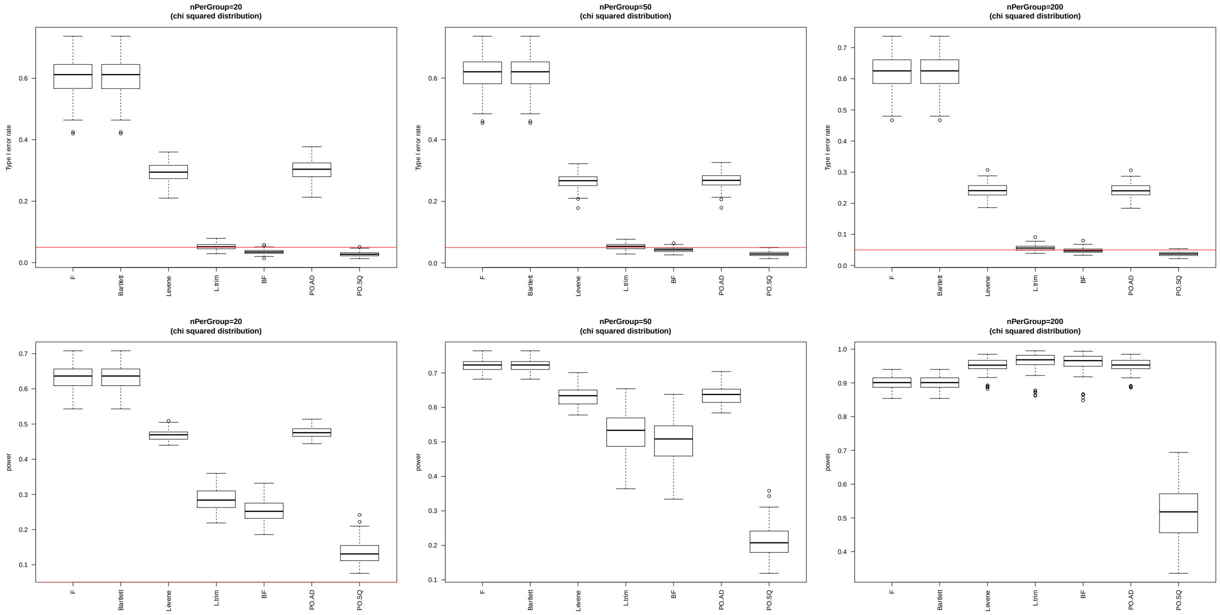

Figure G: Parallel boxplots for scenarios where data were generated from Bayesian hierarchical models with conditional chi squared distributions having different means. Upper panel: Type I error rates (The red horizontal line indicates Type I error rate = 0.05); Lower panel: powers (The red horizontal line indicates power = 0.05). Left panel: 20 subjects per group; middle panel: 50 subjects per group; right panel: 200 subjects per group.

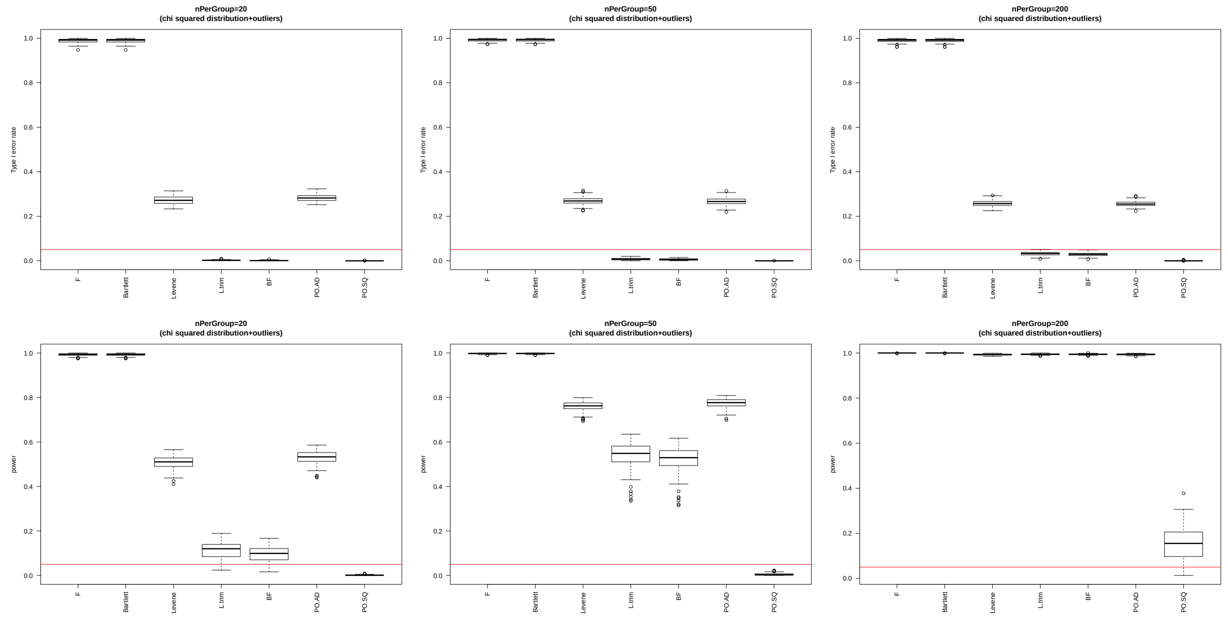

Figure H: Parallel boxplots for scenarios where data were generated from Bayesian hierarchical models with conditional chi squared distributions having different means and outlier. Upper panel: Type I error rates (The red horizontal line indicates Type I error rate = 0.05); Lower panel: powers (The red horizontal line indicates power = 0.05). Left panel: 20 subjects per group; middle panel: 50 subjects per group; right panel: 200 subjects per group.

C.2 Data generated from t distributions

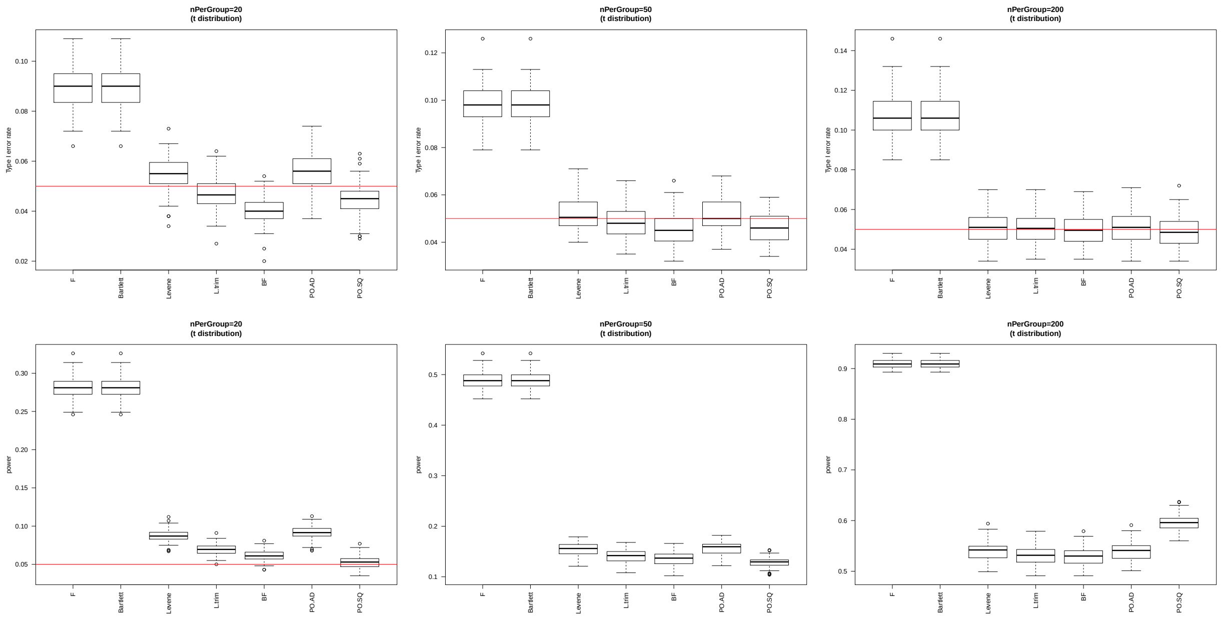

Figure I: Parallel boxplots for scenarios where data were generated from t distributions with equal means. Upper panel: Type I error rates (The red horizontal line indicates Type I error rate = 0.05); Lower panel: powers (The red horizontal line indicates power = 0.05). Left panel: 20 subjects per group; middle panel: 50 subjects per group; right panel: 200 subjects per group.

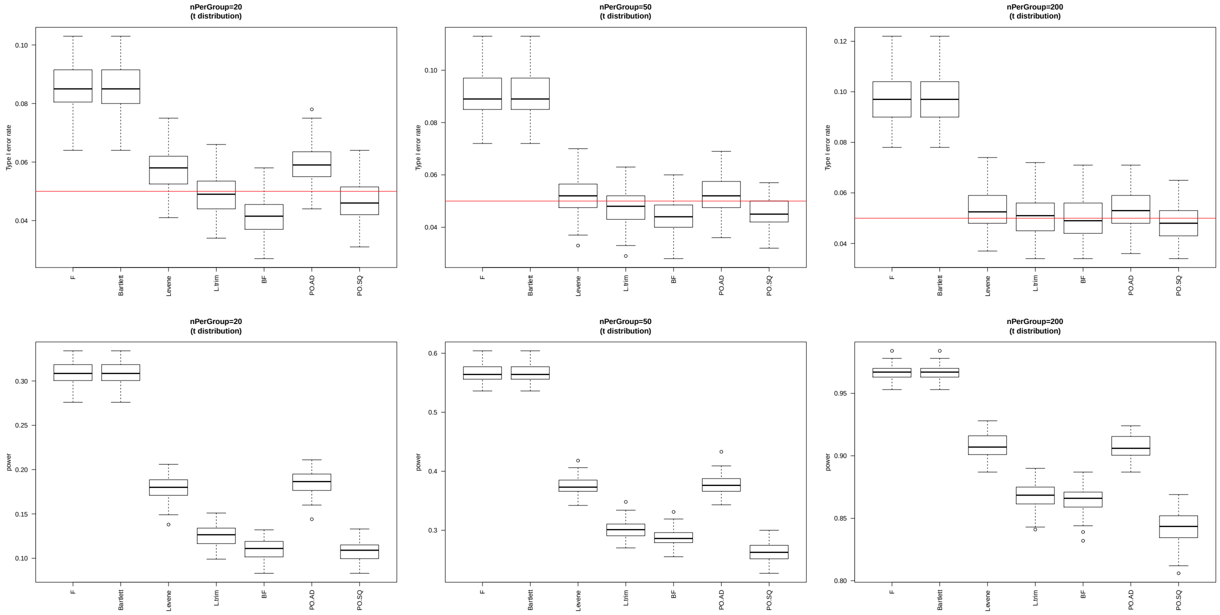

Figure J: Parallel boxplots for scenarios where data were generated from t distributions with different means. Upper panel: Type I error rates (The red horizontal line indicates Type I error rate = 0.05); Lower panel: powers (The red horizontal line indicates power = 0.05). Left panel: 20 subjects per group; middle panel: 50 subjects per group; right panel: 200 subjects per group.

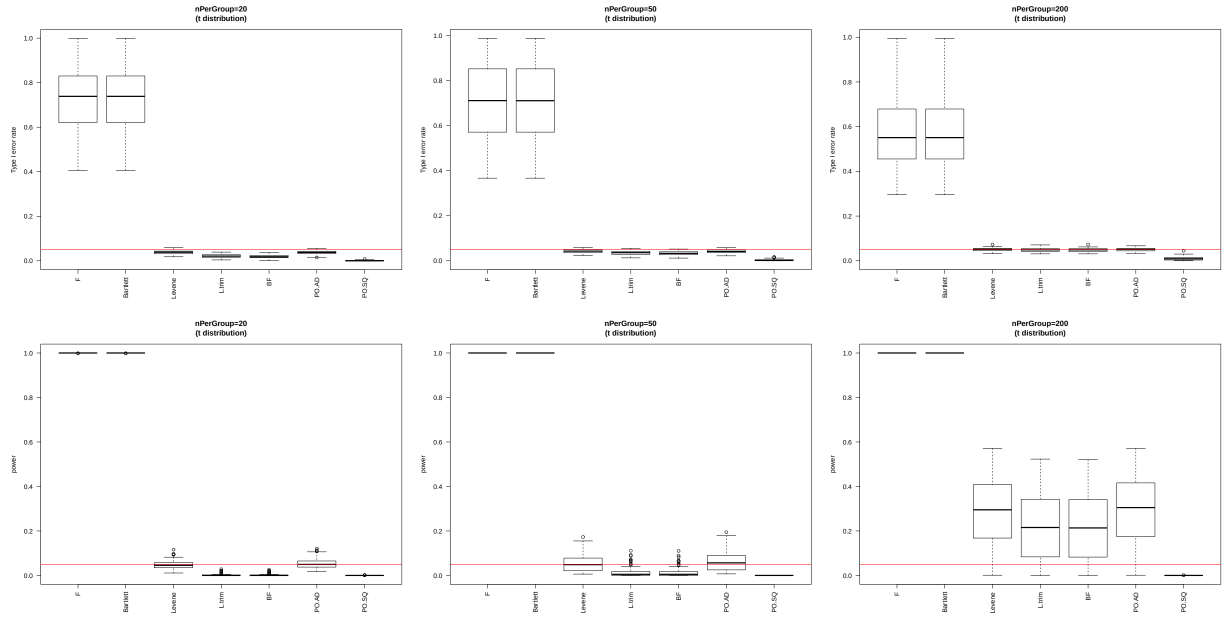

Figure K: Parallel boxplots for scenarios where data were generated from t distributions with equal means and outlier. Upper panel: Type I error rates (The red horizontal line indicates Type I error rate = 0.05); Lower panel: powers (The red horizontal line indicates power = 0.05). Left panel: 20 subjects per group; middle panel: 50 subjects per group; right panel: 200 subjects per group.

C.3 Data generated from (conditional) normal distributions

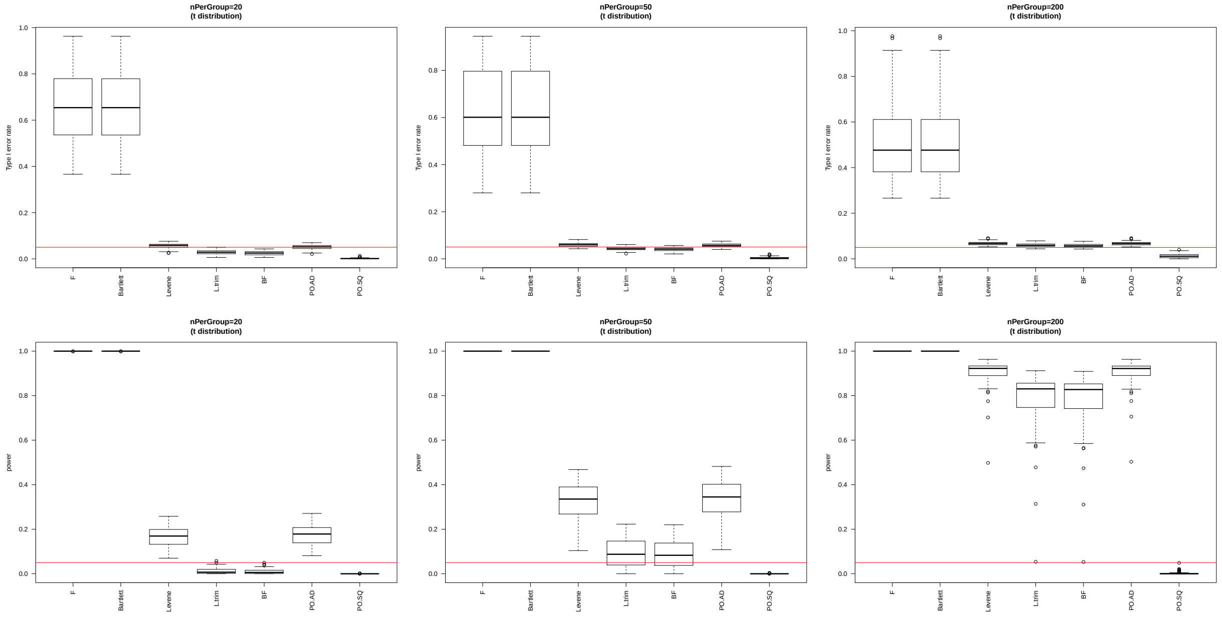

Figure L: Parallel boxplots for scenarios where data were generated from t distributions with different means and outlier. Upper panel: Type I error rates (The red horizontal line indicates Type I error rate = 0.05); Lower panel: powers (The red horizontal line indicates power = 0.05). Left panel: 20 subjects per group; middle panel: 50 subjects per group; right panel: 200 subjects per group.

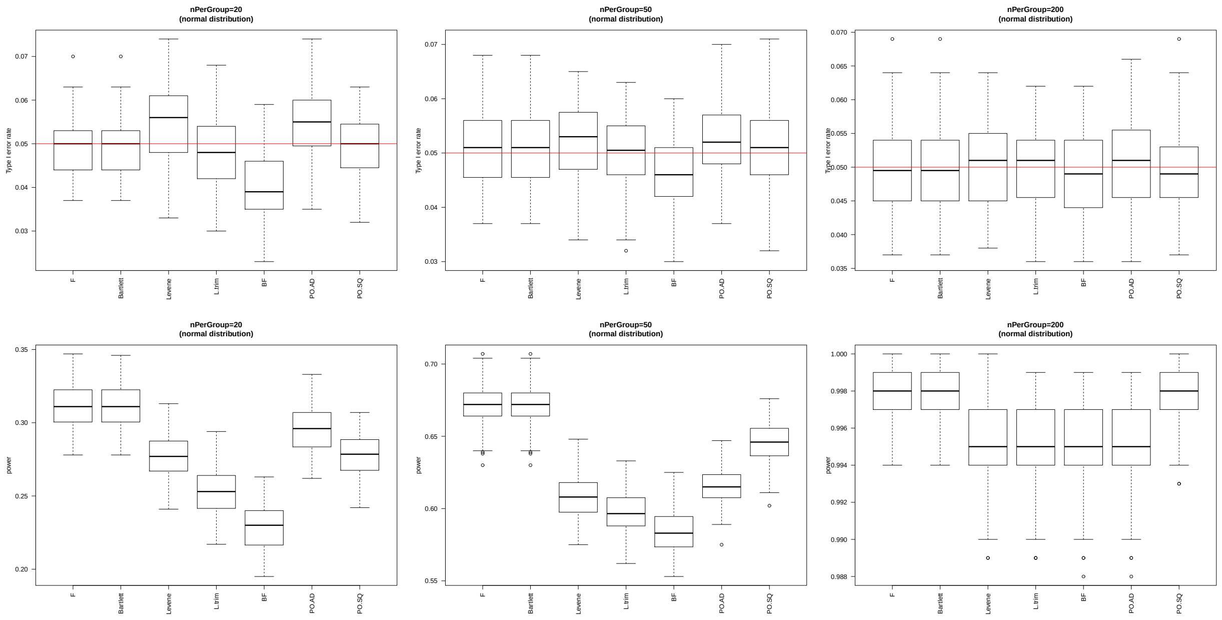

Figure M: Parallel boxplots for scenarios where data were generated from normal distributions with equal means. Upper panel: Type I error rates (The red horizontal line indicates Type I error rate = 0.05); Lower panel: powers. Left panel: 20 subjects per group; middle panel: 50 subjects per group; right panel: 200 subjects per group.

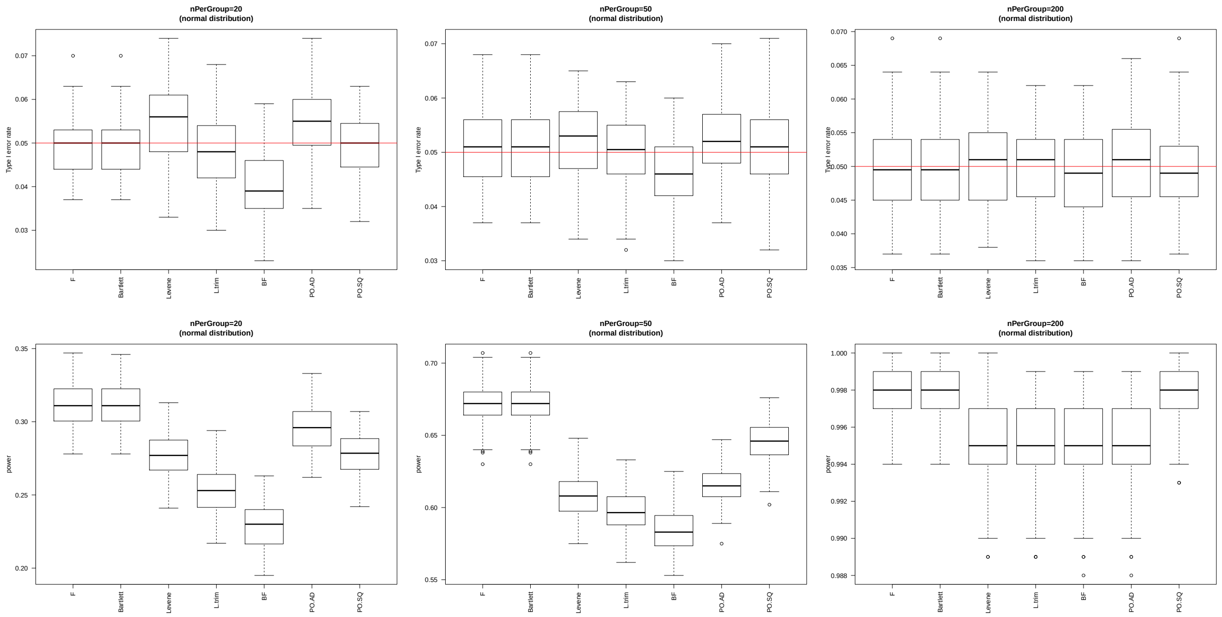

Figure N: Parallel boxplots for scenarios where data were generated from normal distributions with different means. Upper panel: Type I error rates (The red horizontal line indicates Type I error rate = 0.05); Lower panel: powers (The red horizontal line indicates power = 0.05). Left panel: 20 subjects per group; middle panel: 50 subjects per group; right panel: 200 subjects per group.

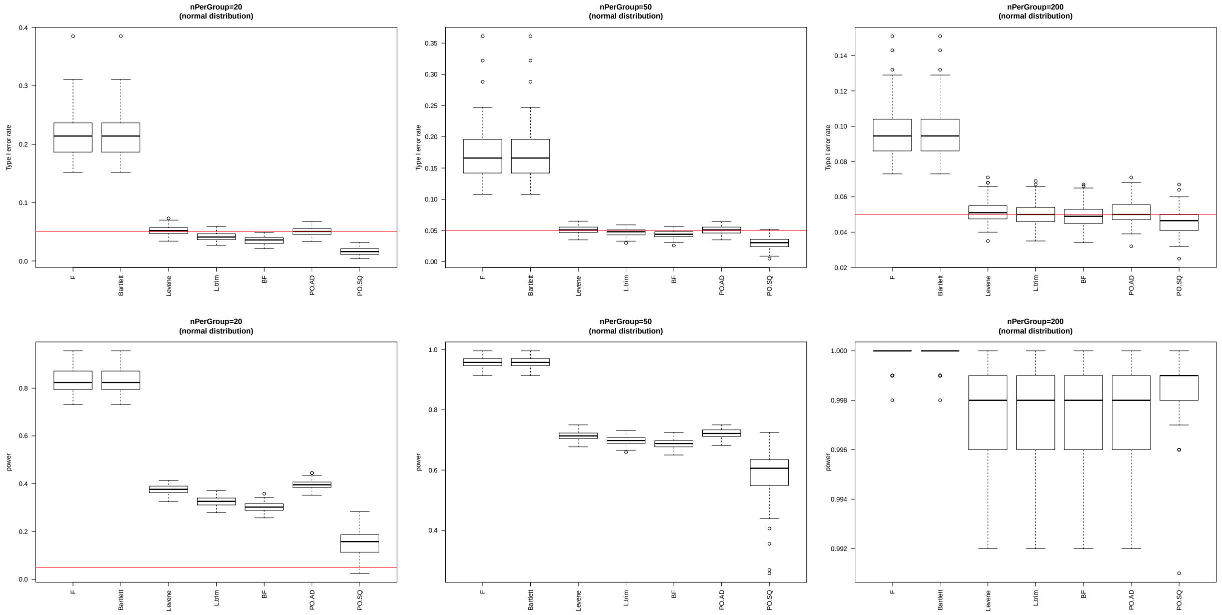

Figure O: Parallel boxplots for scenarios where data were generated from normal distributions with equal means and outliers. Upper panel: Type I error rates (The red horizontal line indicates Type I error rate = 0.05); Lower panel: powers (The red horizontal line indicates power = 0.05). Left panel: 20 subjects per group; middle panel: 50 subjects per group; right panel: 200 subjects per group.

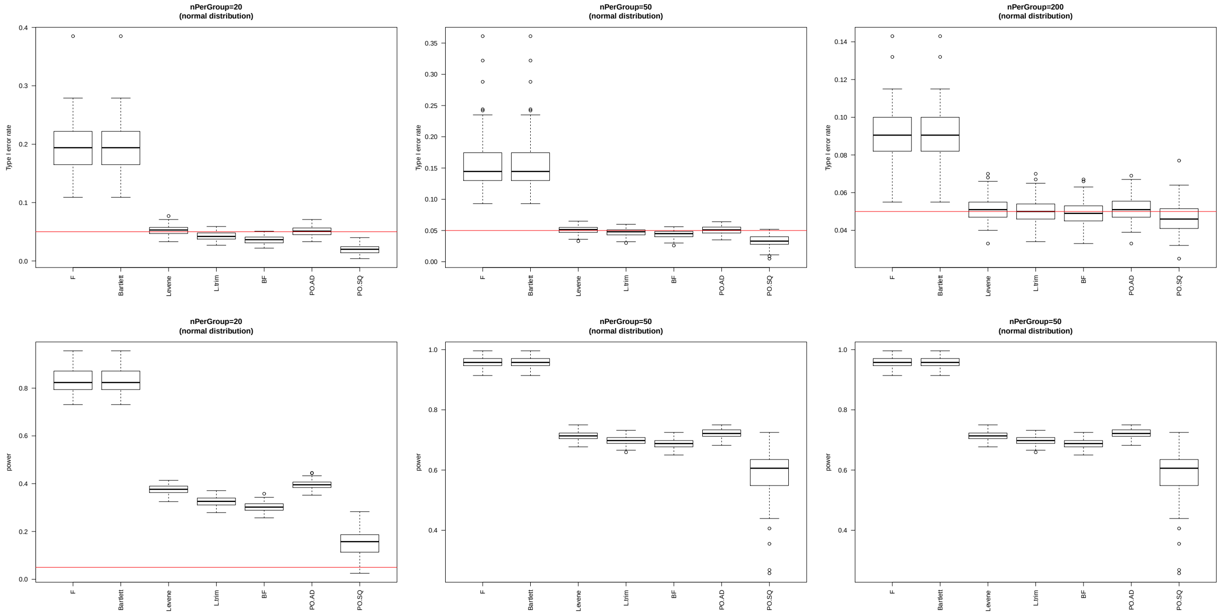

Figure P: Parallel boxplots for scenarios where data were generated from normal distributions with different means and outlier. Upper panel: Type I error rates (The red horizontal line indicates Type I error rate = 0.05); Lower panel: powers (The red horizontal line indicates power = 0.05). Left panel: 20 subjects per group; middle panel: 50 subjects per group; right panel: 200 subjects per group.

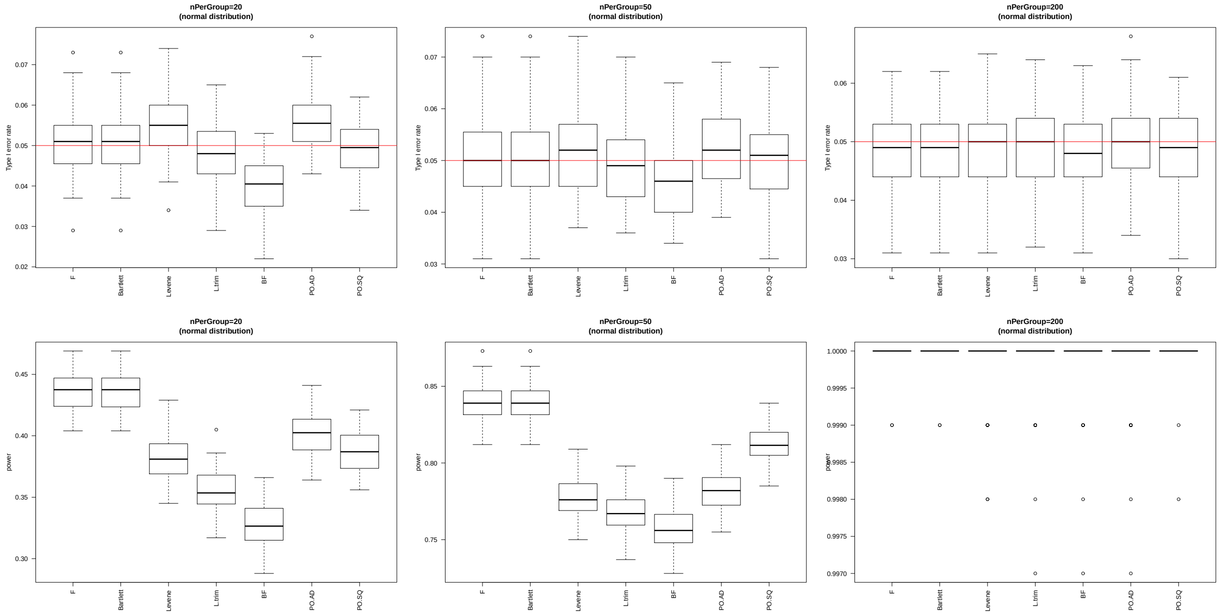

Figure Q: Parallel boxplots for scenarios where data were generated from Bayesian hierarchical models with conditional normal distributions having different means. Upper panel: Type I error rates (The red horizontal line indicates Type I error rate = 0.05); Lower panel: powers (The red horizontal line indicates power = 0.05). Left panel: 20 subjects per group; middle panel: 50 subjects per group; right panel: 200 subjects per group.

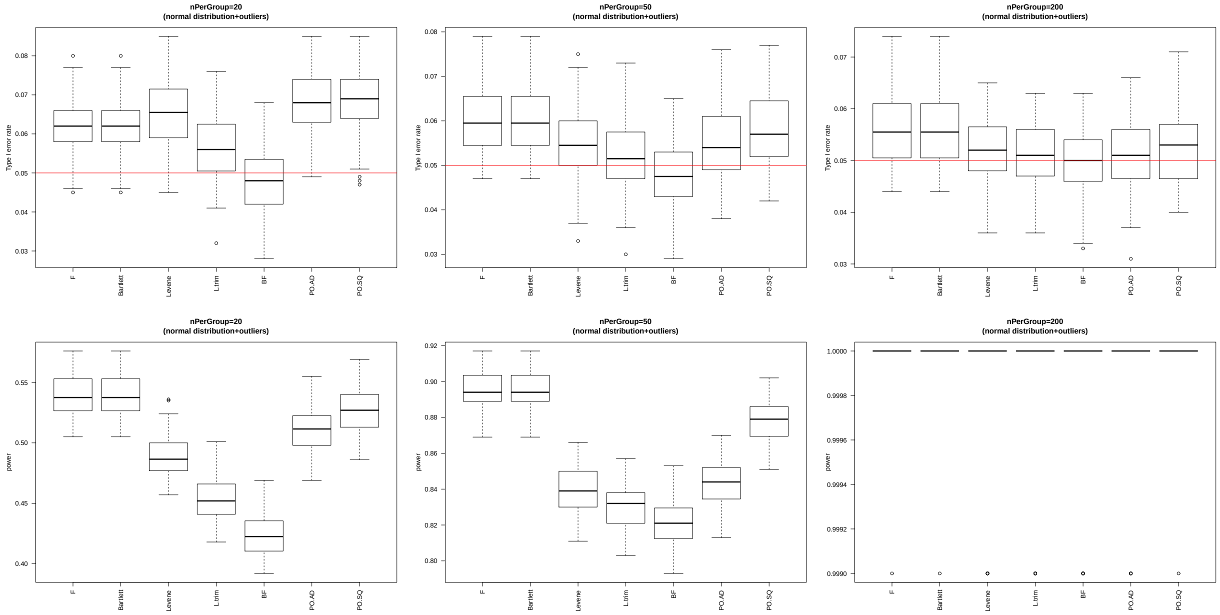

Figure R: Parallel boxplots for scenarios where data were generated from Bayesian hierarchical models with conditional normal distributions having different means and outlier. Upper panel: Type I error rates (The red horizontal line indicates Type I error rate = 0.05); Lower panel: powers (The red horizontal line indicates power = 0.05). Left panel: 20 subjects per group; middle panel: 50 subjects per group; right panel: 200 subjects per group.

D Table of ranks in terms of power

Table A: Ranks of the 7 equal-variance tests in terms of power for each of the 48 simulation scenarios

n	Distr	eqM	out	F	Bartlett	Levene	L.trim	BF	PO.AD	PO.SQ
20	chisq	no	no	-	-	-	-	-	-	-
20	chisq	no	yes	-	-	-	1	2	-	3
20	chisq	yes	no	-	-	-	-	1	-	-
20	chisq	yes	yes	-	-	-	1	2	-	3
20	chisq.c	no	no	-	-	-	-	1	-	2
20	chisq.c	no	yes	-	-	-	1	2	-	3
20	N	no	no	1.5	1.5	-	4	5	-	3
20	N	no	yes	-	-	-	2	3	1	4
20	N	yes	no	1.5	1.5	-	4	5	-	3
20	N	yes	yes	-	-	-	2	3	1	4
20	N.c	no	no	1.5	1.5	-	4	5	-	3
20	N.c	no	yes	-	-	-	-	1	-	-
20	t	no	no	-	-	-	1	2	-	3
20	t	no	yes	-	-	-	2	3	1	4
20	t	yes	no	-	-	-	1	2	-	3
20	t	yes	yes	-	-	2	4	4	1	4
50	chisq	no	no	-	-	-	-	-	-	1
50	chisq	no	yes	-	-	-	1	2	-	3
50	chisq	yes	no	-	-	-	-	1.5	-	1.5
50	chisq	yes	yes	-	-	-	1	2	-	3
50	chisq.c	no	no	-	-	-	-	1	-	2
50	chisq.c	no	yes	-	-	-	1	2	-	3
50	N	no	no	1.5	1.5	-	4	5	-	3
50	N	no	yes	-	-	-	2	3	1	4
50	N	yes	no	1.5	1.5	-	4	5	-	3
50	N	yes	yes	-	-	2	3	4	1	5
50	N.c	no	no	1.5	1.5	-	4	5	-	3
50	N.c	no	yes	-	-	-	-	1	-	-
50	t	no	no	-	-	-	1	2	-	3
50	t	no	yes	-	-	-	1	2	-	3
50	t	yes	no	-	-	-	1	2	-	3
50	t	yes	yes	-	-	2	3.5	3.5	1	5
200	chisq	no	no	-	-	-	-	-	-	1
200	chisq	no	yes	-	-	-	1	2	-	3
200	chisq	yes	no	-	-	-	-	1	-	2
200	chisq	yes	yes	-	-	-	1	2	-	3
200	chisq.c	no	no	-	-	-	-	1	-	2
200	chisq.c	no	yes	-	-	-	1.5	1.5	-	3
200	N	no	no	2	2	5.5	5.5	5.5	5.5	2
200	N	no	yes	-	-	-	2.5	2.5	-	1
200	N	yes	no	2	2	5.5	5.5	5.5	5.5	2
200	N	yes	yes	-	-	-	2.5	2.5	-	1
200	N.c	no	no	4	4	4	4	4	4	4
200	N.c	no	yes	-	-	-	2	2	2	-
200	t	no	no	-	-	-	1	2	-	3
200	t	no	yes	-	-	-	-	-	-	1
200	t	yes	no	-	-	2	4	5	3	1
200	t	yes	yes	-	-	2	3	4	1	5
n_{reject}				39	39	40	12	4	35	5
m				1.5	1.5	2.0	2.0	2.0	1.0	3.0

"-" indicates the null hypothesis H_0 that the type I error rate of the equal-variance test is ≤ 0.05 was rejected; n_{reject} = number of scenarios where a equal-variance test rejected H_0; m = median of the ranks of powers. For ranks with ties, average ranks were used. N.c and chisq.c indicate conditional normal and chi squared distributions, respectively.

References

[1] Phipson, b. and Oshlack, A. DiffVar: a new method for detecting differential variability with application to methylation in cancer and aging. *Genome Biology*, 15:465, 2014.

[2] Smyth, G.K. Linear models and empirical bayes methods for assessing differential expression in microarray experiments. *Statistical Applications in Genetics and Molecular Biology*, 3(1):Article 3, 2004.
